# Comparison of erectile and ejaculatory functional outcomes between unilateral and bilateral cavernosal rupture in penile fractures

**DOI:** 10.1038/s41443-024-00940-4

**Published:** 2024-06-25

**Authors:** Emin Taha Keskin, Osman Can, Yiğit Can Filtekin, Harun Özdemir, Mehmet Şahin, Gökhan Çeker, Cemal Topal, Halil Lütfi Canat

**Affiliations:** Department of Urology, Basaksehir Cam and Sakura City Hospital, Istanbul, Turkey

**Keywords:** Erectile dysfunction, Sexual dysfunction

## Abstract

This study aimed to compare the erectile and ejaculatory functional outcomes of unilateral and bilateral ruptures of the corpus cavernosum in penile fractures. Sixty patients’ data were analyzed retrospectively between June 2020 and January 2023. The patients were divided into two groups based on the affected corpus cavernosum (unilateral and bilateral). Preoperative and postoperative 3rd-, 6th-, and 12th-month self-estimated intravaginal-ejaculation-latency-time (IELT), and international index of erectile function-erectile function (IIEF-EF) scores as well as the presence of urethral injury were compared. Bilateral corpus cavernosum fractures were detected in 18.3% of the patients. The IIEF-EF scores of both groups at 3rd-, 6th-, and 12th-month were found to be significantly lower than the preoperative scores (unilateral group:24.1 ± 2.7 vs 23.2 ± 3.5 and 23.3 ± 3.4, respectively, p = 0.011 and 0.014, respectively; bilateral group: 24 ± 1.9 vs 23 ± 1.8 and 23.2 ± 1.5, respectively, p = 0.027 and 0.047, respectively). No significant difference was found between the preoperative and the postoperative 12th month IIEF-EF scores in either group (unilateral group: 24.1 ± 2.7 vs 23.4 ± 3.6, p = 0.207;bilateral group:24 ± 1.9 vs 23.2 ± 1.5, p = 0.057). The self-estimated IELTs of both groups at the postoperative 3rd, 6th, and 12th months demonstrated a significant increase from the preoperative values (unilateral group: 221.6 ± 81.8 vs 252 ± 94.6, 256.5 ± 97.6, and 250.5 ± 104.8, respectively, p < 0.001; bilateral group:241.8 ± 61.6 vs 278.1 ± 55.4, 281.8 ± 56.1, and 283.6 ± 54.2, respectively, p = 0.041, 0.030, and 0.047, respectively). The changes in self-estimated IELTs and IIEF-EF scores between the preoperative period and the postoperative 3rd, 6th, and 12th-months were compared, and no statistical difference was found between patients with unilateral and bilateral corpus cavernosum fractures (p > 0.05). In conclusion, no significant difference in erectile function was found in either group at the 12-month follow-up, and the self-estimated IELTs were found to be prolonged in both groups. Furthermore, no difference was noted between the groups at any follow-up. To explain the effects of unilateral and bilateral injuries on erectile and ejaculatory functions, further studies with a larger-number of patients are necessary.

## Introduction

Penile fracture (PF) is defined as the rupture of the tunica albuginea of the corpus cavernosum [1]. The incidence of PF is relatively low, with a prevalence of approximately 1 in 100,000 in the USA [2].

PF occurs especially during sexual intercourse, but it can also be a consequence of any type of trauma [3]. In Western nations, PF is commonly diagnosed as a result of the erect penis hitting the female pelvis during sex. However, in Eastern countries, the “*Taghaandan*” manoeuvre and traumatic masturbation appear to be responsible for a significant number of cases [4]. The diagnosis of PF is primarily based on clinical history. Penile hematoma is known as the most common finding [5]. The patient describes pain, quick detumescence, swelling, and bruising after hearing a cracking sound from the corpus cavernosum [6]. The rupture of the corpus cavernosum is a urological emergency; it can lead to erectile and ejaculatory functional changes in the long-term. To prevent these complications, emergency exploration is accepted as an appropriate treatment for PF [7]. However, no statistically significant correlation has been found between the timing of PF repair and the incidence of complications [8].

In PF, unilateral, rather than bilateral, corpus cavernosum laceration is more common, and it is believed that a PF associated with bilateral corporal lacerations indicates high-energy trauma [9]. Therefore, we hypothesised that the post-surgical erectile and ejaculatory functions of a patient with PF and bilateral corpus cavernosum damage due to high-energy trauma may differ from those of a patient with PF and unilateral corpus cavernosum damage due to low energy trauma.

To the best of our knowledge, this is the first study to compare the sexual and functional outcomes of unilateral and bilateral ruptures of the corpus cavernosum in PFs.

## Materials and methods

In this retrospective analysis, we collected data from patients diagnosed with and undergoing immediate surgical repair for PF at our high volume reference hospital between June 2020 and January 2023 after obtaining institutional review board approval (KAEK/2023.05.215).

After excluding PF caused by reasons other than sexual intercourse (such as gunshot injury), a total of 71 patients were included in our study. Patients with surgery performed more than 12 hours after injury (*n* = 2), less than six months of follow-up (*n* = 6), and missing data (*n* = 3) were excluded from the study. Therefore, data from a total of 60 patients were used for statistical analysis.

All the patients underwent surgery within 12 hours. A circumferential incision was used proximal to the coronal sulcus to facilitate a full degloving of the penis. After identifying cavernosal (unilateral or bilateral) and urethral injuries, the tunica and urethra were repaired with absorbable sutures. Following surgical repair, if there was a doubt about the stability of the repair or missed cavernosal injuries, artificial penile tumescence was induced by placing a Penrose drain around the base of the penis with a clamp to act as a tourniquet and using a 22 G butterfly needle placed laterally in one corporal body to inject saline into the corpora. If urethral repair was performed due to urethral damage, an indwelling Foley catheter was left for 7–10 days after surgery. Each patient’s perioperative data was evaluated, and the status of the affected corpus cavernosum (unilateral or bilateral) as well as the urethral-injury status were recorded during surgery. Sexual activity was not allowed for six weeks after the surgery for all patients.

Preoperative self-estimated intravaginal ejaculation latency time (IELT) [10–12], and international index of erectile function - erectile function (IIEF-EF) scores [13–15] were recorded just before surgery for all the patients. Postoperative (3, 6, and 12 months) self-estimated IELTs and IIEF-EF scores were recorded for all the patients at our andrology outpatient clinic. Both the preoperative and postoperative self-estimated IELTs and IIEF-EF scores were recorded according to the medical history of the patient and his partner. In the presence of postoperative penile curvature, 30 degrees and above were considered significant. The presence of postoperative palpable nodule was noted. Patients who were unable to attend follow-ups were contacted via telephone to determine whether they had experienced any complications.

### Statistical analysis

In this study, data obtained from personal information forms and scales were transferred to a computer and analyzed via SPSS (Statistical Package Programme for Social Sciences 22.0) program. The patients were divided into two groups according to the status of the affected corpus cavernosum (unilateral and bilateral). Parameters for the distribution of continuous variables (preoperative and postoperative 3rd-, 6th-, and 12th-month self-estimated IELTs and IIEF-EF scores) were calculated, and their distributions were tested for normality using the Kolmogorov-Smirnov test. T-Test and Mann-Whitney-U were used for these parameters to compare groups. To compare the quantitative variables before and after PF, a paired t test was used for normally distributed data, while the Wilcoxon test was used for data not normally distributed. The p-value was accepted <0.05 at a 95% confidence interval.

## Results

Bilateral corpus cavernosum fracture was detected in 18.3% (*n* = 11), and urethral injury was found in 20% (*n* = 12) of all patients. A total of 83.3% (*n* = 10) of all patients with concomitant urethral injuries (*n* = 12) were found in the bilateral cavernosal fracture group. A postoperative palpable nodule was found in 65% of patients at 12 months. Neurovascular bundle injury was not found in any of the patients. No clinically significant penile curvature of 30 degrees or more was detected in any of the patients. No erectile dysfuncton (ED) was reported, and no one required therapy for ED during follow-up. The mean preoperative IIEF-EF scores and self-estimated IELTs were found 24.1 ± 2.6 and 229 ± 79.6, respectively (Table 1).

**Table 1:** Demographic data and preoperative sexual functions.

	All patients (n:60)
*Side of Penile Fracture % (n)*	
Unilateral	81.7% (49)
Right	45% (27)
Left	36.7% (22)
Bilateral	18.3 (11)
Urethral injury % (*n*)	20% (12)
Unilateral	16.7% (2)
Bilateral	83.3% (10)
Postoperative palpable nodule % (*n*)	65% (39)
Preoperative Sexual Functions (mean ± SD)	
IIEF-EF score	24.1 ± 2.6
Self-estimated IELT (sec.)	229 ± 79.6

*IIEF-EF* International Index of Erectile Function - Erectile function (IIEF-EF), *IELT* Intravaginal Ejaculation Latency Time, *Sec.* Second.

**Fig. 1:**
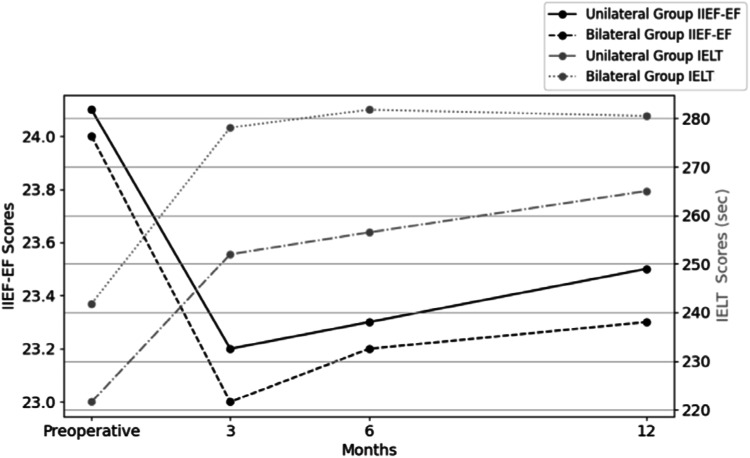
Self-estimated IELTs and IIEF-EF scores of unilateral and bilateral groups. International Index of Erectile Function - Erectile function (IIEF-EF); IELT: Intravaginal Ejaculation Latency Time; Sec: Second.

When analysing the IIEF-EF scores of all the patients (*n* = 60), a statistically significant decrease was found at three and six months after surgery in compared to the preoperative period (23.2 ± 3.3 and 23.3 ± 3.1 vs 24.1 ± 2.6, respectively; p = 0.019 and 0.003, respectively). However, this difference was not statistically significant when comparing the postoperative score at 12 month to the preoperative one for all patients (23.3 ± 3.3 vs 24.1 ± 2.6, respectively; p = 0.170). The IIEF-EF scores at three and six months of both the unilateral and bilateral groups were found to be significantly lower than the preoperative score. In the unilateral group, the scores were 23.2 ± 3.5 and 23.3 ± 3.4 vs 24.1 ± 2.7, respectively (p = 0.011 and 0.014, respectively). In the bilateral group, the scores were 23 ± 1.8 and 23.2 ± 1.5 vs 24 ± 1.9, respectively (p = 0.027 and 0.047, respectively). No significant difference was found between the preoperative and postoperative 12 months IIEF-EF scores in the two groups (unilateral group: 24.1 ± 2.7 vs 23.4 ± 3.6, p = 0.207; bilateral group: 24 ± 1.9 vs 23.2 ± 1.5, p = 0.057) (Table 2, Fig. 1). No statistically significant difference was found between the unilateral and bilateral groups when comparing the changes in IIEF-EF scores at the 3, 6, and 12 months to the preoperative score (p = 0.836, 0.977, and 0.645, respectively) (Table 3).

**Table 2:** Preoperative and postoperative self-estimated IELTs and IIEF-EF scores of unilateral and bilateral fractures.

			Mean ± SD	Min - Max	*p* value
All Patients (n:60)	IIEF-EF Score	**Preoperative**	24.1 ± 2.6	17–30	Ref.
		**Postoperative**			
		3rd month	23.2 ± 3.3	16–30	0.019*
		6th month	23.3 ± 3.1	15–30	0.003**
		12th month	23.3 ± 3.3	17–30	0.170*
	IELT (sec.)	**Preoperative**	229 ± 79.6	150–640	Ref.
		**Postoperative**			
		3rd month	256.8 ± 88.9	150–700	<0.001*
		6th month	261.1 ± 91.8	150–720	<0.001*
		12th month	258.4 ± 95.7	150–760	<0.001*
Unilateral (n:49)	IIEF-EF Score	**Preoperative**	24.1 ± 2.7	17–30	Ref.
		**Postoperative**			
		3rd month	23.2 ± 3.5	16–30	0.011*
		6th month	23.3 ± 3.4	15–30	0.014*
		12th month	23.4 ± 3.6	17–30	0.207*
	IELT(sec.)	**Preoperative**	221.6 ± 81.8	150–640	Ref.
		**Postoperative**			
		3rd month	252 ± 94.6	150–700	<0.001*
		6th month	256.5 ± 97.6	150–720	<0.001*
		12th month	250.5 ± 104.8	150–760	<0.001*
Bilateral(n:11)	IIEF-EF Score	**Preoperative**	24 ± 1.9	21–26	Ref.
		**Postoperative**			
		3rd month	23 ± 1.8	17–25	0.027**
		6th month	23.2 ± 1.5	17–25	0.047**
		12th month	23.2 ± 1.5	19–27	0.057**
	IELT(sec.)	**Preoperative**	241.8 ± 61.6	180–360	Ref.
		**Postoperative**			
		3rd month	278.1 ± 55.4	240–420	0.041**
		6th month	281.8 ± 56.1	220–420	0.030**
		12th month	283.6 ± 54.2	240–420	0.047**

*IIEF-EF* International Index of Erectile Function - Erectile function (IIEF-EF), *IELT* Intravaginal Ejaculation Latency Time, *Max:* Maximum, *Min:* Minimum, *Sec.* Second.

*Wilcoxon test; **Paired *T* test.

Statistically significant *p*-values are in bold.

**Table 3:** Comparison of the change in self-estimated IELTs and IIEF-EF scores between preoperative and postoperative values at 3, 6, and 12 months for bilateral and unilateral fractures.

		Unilateral (n:49)	Bilateral (n:11)	*p* value
		Mean ± SD	Mean ± SD	
Δ IIEF-EF Score	Preop. vs 3. months	−0.9 ± 3	−0.9 ± 1.8	0.836*
	Preop. vs 6. months	−0.8 ± 3.2	−0.7 ± 1.2	0.977*
	Preop. vs 12. months	−0.6 ± 4	−1.18 ± 1.1	0.645**
	3. vs 6. months	0.02 ± 1.3	0.18 ± 1.8	0.857*
	6. vs 12. months	0.03 ± 1.3	−0.45 ± 0.9	0.349*
Δ IELT (sec.)	Preop. vs 3. months	30.4 ± 40.3	26.3 ± 54.2	0.332**
	Preop. vs 6. months	34.9 ± 40.9	30 ± 60.6	0.325**
	Preop. vs 12. months	32.8 ± 43.5	31.8 ± 61.6	0.512**
	3. vs 6. months	4.4 ± 14.2	3.6 ± 19.6	0.396*
	6. vs 12. months	6.2 ± 15.9	1.8 ± 6.1	0.503*

*IIEF-EF* International Index of Erectile Function - Erectile function (IIEF-EF), *IELT* Intravaginal Ejaculation Latency Time, *Sec.* Second.

*Mann–Whitney-*U*, **t Test.

With regard to ejaculatory function, all the patients’ postoperative self-estimated IELTs (at 3, 6, and 12 months) were statistically significantly longer than their preoperative scores (*p* < 0.001). Both the unilateral and bilateral groups demonstrated significant increases in their IELTs at 3, 6, and 12 months after surgery compared to their preoperative values (unilateral group: 252 ± 94.6, 256.5 ± 97.6, and 250.5 ± 104.8 vs 221.6 ± 81.8, respectively, *p* < 0.001; bilateral group: 278.1 ± 55.4, 281.8 ± 56.1, and 283.6 ± 54.2 vs 241.8 ± 61.6, respectively, *p* = 0.041, 0.030, and 0.047, respectively) (Table 2).

The difference in the changes between the preoperative and postoperative (3, 6, and 12 months) self-estimated IELTs was compared in the unilateral and bilateral groups, and no statistical difference was found (*p* > 0.05) (Table 3).

The difference in the changes between the preoperative and postoperative 3, 6, and 12 months IIEF-EF and self-estimated IELTs were compared based on the presence of urethral injury, and no statistical difference was found (*p* > 0.05) (Table 4).

**Table 4:** Comparison of the change in self-estimated IELTs and IIEF-EF scores according to presence of urethral injury at preoperative, 3, 6, and 12 months for penile fractures.

		Urethral Injury		*p* value
		No (n:48)	Yes (n:12)	
		Mean ± SD	Mean ± SD	
Δ IIEF-EF Score	Preop. vs 3. months	−0.9 ± 3.1	−0.9 ± 1.7	0.760*
	Preop. vs 6. months	−0.9 ± 3.2	−0.67 ± 1.3	0.910*
	Preop. vs 12. months	−0.6 ± 4.1	−1.08 ± 1.3	0.705**
	3. vs 6. months	0.01 ± 1.3	0.25 ± 1.8	0.790*
	6. vs 12. months	0.03 ± 1.3	−0.42 ± 0.7	0.349*
Δ IELT (sec.)	Preop. vs 3. months	33.5 ± 35.4	5 ± 62.1	0.059**
	Preop. vs 6. months	37.2 ± 38.4	11.6 ± 62.9	0.077**
	Preop. vs 12. months	36.7 ± 38.7	11.6 ± 66.3	0.120**
	3. vs 6. months	3.7 ± 13.4	6.6 ± 21.4	0.888*
	6. vs 12. months	7.1 ± 15.4	0.01 ± 8.5	0.143*

*IIEF-EF* International Index of Erectile Function - Erectile function (IIEF-EF), *IELT* Intravaginal Ejaculation Latency Time, *Preop* Preoperative value, *Sec.* Second.

*Mann–Whitney-*U*, **t Test.

## Discussion

A PF is a type of traumatic penile injury. During flaccidity, the thickness of the tunica albuginea is 2 mm, which decreases to 0.25–0.50 mm during erection. It is not unexpected that 80% of ruptures are transverse and ventral, given that the ventral side of the corpus cavernosum is the thinnest area [16]. Immediate detumescence, oedema, and hematoma after hearing a cracking sound are the well-known early symptoms of the rupture of the corpus cavernosum. At the time of the PF, none of these symptoms indicate whether the corpus cavernosum was affected bilaterally or unilaterally [17]. To the best of our knowledge, there is no study comparing postoperative erectile- and ejaculatory-function changes after unilateral and bilateral corpus cavernosum fractures.

The degree of penile trauma influences the size of a tunica albuginea defect, which can occur with fracture and other complications. A PF with bilateral corporal lacerations is thought to indicate high-energy trauma. Urethral damage may also occur in the presence of bilateral corporal lacerations due to such kind of trauma [18]. In the literature, the rate of bilateral cavernosal rupture after PF is reported to range between 0% and 26.3%, while urethral damage occurs in 5.8–27.5% of cases [9, 19]. In this study, bilateral corpus cavernosum rupture was found in 18.3% of patients with PF, and urethral injury was detected in 20% of patients.

One of the important complications of PF is known as ED. The prevalence of ED after a PF ranges from 0% to 34.6% [20]. Some studies have shown that ED may occur after surgically treated PFs in the long term. Barros et al. and Hatzichristodoulou et al. found that the rate of ED after surgical intervention was reported to be 13.1% and 30.8%, respectively [8, 21]. Other studies showed that ED rates were very low (1.4%) after surgical treatment of PF, but that they rates were significantly higher (80%) after conservative treatment. In a few studies, ED development after PF repair was not reported [22].

We analysed patients’ pre- and post-operative erectile functions using the IIEF-EF questionnaire. Erectile functions were not different from the preoperative period at the 12-month follow-up, even though a statistically significant decrease was found three and six months after surgical repair in both the unilateral and bilateral corpus cavernosum fracture groups. No statistically significant differences were found between the two groups when comparing preoperative and postoperative erectile function changes. In other words, a bilateral corpus cavernosum fracture did not worsen postoperative erectile function compared to a unilateral fracture.

In the literature, data on ejaculatory function after PF is limited. Therefore, the pathophysiology of prolonged ejaculation time following PF is unclear. Only a few studies have shown that ejaculatory dysfunction can occur after PF. In the study of Barros et al., delayed ejaculation disorder was found to be more common, and premature ejaculation was found to be rare (it was observed in only one patient) [7]. In another study, postoperative IELTs were found to be statistically higher than the preoperative scores in patients who had suffered PFs [23]. In our study, almost all the patients showed increases in self-estimated IELTs after surgical repair compared to the preoperative period. The IELT values at 3, 6, and 12 months after surgical treatment were found to be significantly higher than the preoperative scores. This statistically significant increase in self-estimated IELTs was found in both the unilateral and bilateral groups. These results, show that the prolongation of ejaculation times actually occured as a result of the PFs.

Different causes may explain ejaculatory changes after PF. First, a rupture of the corpus cavernosum may result in neurovascular damage, which could affect penile sensation. To demonstrate a neurogenic injury that may affect erectile function and ejaculation time, comprehensive neurologic evaluations are required. Second, failure to reach satisfactory rigidity may lead to a delay in reaching orgasm or even a prolongation in ejaculation time, but there is no data in the literature that supports this hypothesis. In our study, the decrease in erectile function in both the unilateral and bilateral groups at 3 and 6 months after surgery might have led to a prolongation in ejaculation time. However, 12 months, although patients’ erectile functions became similar to the preoperative period, the detected prolongation in the ejaculation times continued. This result suggests that there might be another underlying mechanism at play. PF-related postoperative psychological changes could lead to differences in ejaculatory time [24]. Another hypothesis is that the tunica albuginea is thicker on the dorsal side than on the ventral side. For this reason, PF related to high-energy trauma is frequently found in the ventral tunica albuginea [16]. Due to the distance of the neurovascular bundle from the ventral side, ejaculatory functions may be preserved in bilateral corpus cavernosum fractures.

In the literature, urethral damage is found in 5.8–27.5% of PFs [9]. Bleeding at the urethral meatus might be the first sign of urethral injury in only 5.6% of cases [25]. Invasive diagnostic tools are not required to evaluate the urethra, as the injury tends to be located at the same level as the corporal tear. Urethral repair with absorbable sutures is recommended during early surgery, along with the use of a long urethral catheter [26]. There is insufficient data on the long-term effect of urethral damage on erectile and ejaculatory functions. In the present study, we found urethral damage in 20% of the patients. According to our findings, urethral damage was found in 91% of patients with bilateral corpus cavernosum fractures. We also showed that the presence of urethral damage had no effect on the changes in erectile and ejaculatory functions compared to the preoperative period in the long-term.

Early surgical repair is recommended to reduce the risk of sexual and erectile complications after PF [27, 28]. There is currently no valid cut-off time for surgery to prevent complications. Bulbul et al. found that with earlier (<13.5 hour) surgical treatment of PFs, postoperative complication rates were lowered [29]. However, in another study, there was no difference between patients admitted to the hospital within 24 hours of the trauma and patients admitted after 24 hours in terms of complications [30]. Since there is no clear cut-off period in the literature, we only included patients who underwent surgical repair within the first 12 hours after injury in order to minimise the potential effect of difference in surgery time.

Most studies have focused on the association between erectile and ejaculatur dysfunctions and PF. However, PFs can have other long-term effects. Several studies have found that the rate of penile deviation after surgical intervention in patients with PF ranged from 2.8% to 11.5% [31]. In a multicentre study, the rate of surgical intervention for penile deviation after PF was found to be 2.2% of patients [32]. In our study, no significant penile deviation due to PF was observed at follow-up. Another complication of PF is palpable fibrotic nodules. In the present study, such nodules were detected in 65% of patients. In Baros et al.’s study though palpable fibrotic nodules were observed in the operative area in all cases [7].

Our study has some limitations. First, the small number of patients and the retrospective study design are the primary limitations of this study. Second, the corpus cavernosum damage was noted by the surgeon during the operation; radiological imaging was not routinely used to support this evidence. Due to this missing data, the effects of tear size and location of the injured area (dorsal, ventral, or lateral) on complications could not be evaluated. Third, the effects of urethral injuries on patients’ erectile and ejaculatory functions could not be compared in the unilateral and bilateral groups due to the insufficient number of patients in the former. Fourth, the results of the study may change if the surgery cut-off time is altered.. Due to the lack of the cut-off time, we only included patients who were operated on within 12 hours of their PFs. Although this is a limitation, early surgical repair could also be considered a strength of our study, as it prevented the risk of increased complications with delayed repair. Fifth, measuring IELTs with a calibrated stopwatch would have been more objective, but since our study was retrospectively designed, both pre- and post-operative self-estimated IELTs and IIEF-EF scores were recorded according to the medical history of the patient and his partner. The use of self-reporting might have caused the self-estimated IELTs to differ from the true value.

## Conclusion

Penile fracture, a type of traumatic penile injury, can cause erectile and ejaculatory function changes after rupture of the corpus cavernosum. In this study, we showed that long-term erectile functions did not change in the presence of either unilateral or bilateral corpus cavernosum fractures. Furthermore, the ejaculation time was found to be prolonged compared to the preoperative period. The effects of unilateral and bilateral injuries on erectile and ejaculatory function may be better explained by further studies involving a larger number of patients.

## Data Availability

The datasets generated during and analysed during the current study are available from the corresponding author on reasonable request.
